# Meningeal TB in a 39-Year-Old Male Presenting with Headache

**DOI:** 10.1155/2017/4753670

**Published:** 2017-09-26

**Authors:** Jason Selinger, Thar El Baage

**Affiliations:** Southeastern Regional Medical Center, Lumberton, NC, USA

## Abstract

Meningeal tuberculosis is rare in the developed world, with only 92 cases of meningeal TB reported in the United States in 2014 according to the CDC (CDC, 2015). We describe the case of a 39-year-old male with a history of alcohol abuse, whose cerebral spinal fluid acid fast smears confirmed TB only weeks after his death. His only initial presenting symptom was headache, and his condition declined rapidly during his hospitalization.

## 1. Intro/Background

*Mycobacterium tuberculosis* can produce several extrapulmonary manifestations, including hematogenous spread to just about anywhere in the body. Examples include spread to joints, bones, the GI system, the cardiovascular system, and the central nervous system. Once in the central nervous system, several forms of the disease may appear, including a tuberculoma which may lead to meningeal infection, and the disease may spread to the spinal cord [1].

According to recent CDC data from 2014, CNS tuberculosis accounted for 4.5% of all extrapulmonary manifestations of TB in the United States [2]. Initial presenting symptoms of meningeal TB may occur in several phases and in different forms. Patients may experience headache, malaise, or fevers early in the disease and may progress to more focal neurological findings as the disease progresses [3].

These symptoms, while vague and often nonspecific, are presented in a variety of smaller review articles with similar findings. Only a small number of patients are aware of a personal history of TB infection [4]. However, adults at higher risk of meningeal TB infection from reactivation of a latent infection include those with a history of immunosuppression from infection, alcohol abuse, or medications. As the disease progresses, a hydrocephalus may occur secondary to inflammation from the surrounding infection and may be viewed as a poor prognostic indicator [5]. Prompt treatment with appropriate antituberculous drugs is critical in preventing morbidity and mortality.

## 2. Case

A 39-year-old male with a past medical history of alcohol abuse of several years' duration presented to the Emergency Department with chief complaint of headache. At the time of presentation he denied any chest pain, weakness, or other neurologic symptoms. Basic labs including complete blood count and basic metabolic panel were performed in the ED, which showed severe hyponatremia with a sodium of 116 mmol/L (normal 135–145 mmol/L) and serum osmolality of 235 mmol/L (normal 275–285 mmol/L). Further questioning of a family member revealed the patient was also experiencing mild confusion in the past few days. Physical exam showed the patient was oriented to person and place, while showing signs of chronic malnutrition. The rest of his serum chemistries, including complete blood count, were normal on presentation with the exception of hypochloremia (81 mmol/L). CT of the head on admission was obtained due to the patient's altered mental status, and it showed no edema or signs of infarct at the time. His chest X-ray performed in the ED showed nonspecific increased reticular markings. Kernig's and Brudzinski's signs were negative on physical exam. The patient had no pain or stiffness of the neck on admission. His vitals were otherwise stable, with normal temperature, O2 saturation, and respiratory rate on presentation. Alcohol level was negative in the ED, although he was kept on alcohol withdrawal protocol for the duration of his hospital stay due to the history of alcohol abuse and likelihood for withdrawal. The patient was admitted to a medical floor for hyponatremia and alcohol withdrawal workup. He did not receive any antibiotics in the ED as there was no indication of an infection at the time.

On the first day of admission, the patient was difficult to arouse in the morning after he spiked a fever of 101.5 F (38.6 C) overnight. Infectious Disease was consulted due to concern for meningitis due to altered mental status and fever. ID recommended starting the patient on Vancomycin, Zosyn, and Acyclovir, which the patient continued for the duration of his two-week hospital stay. A lumbar puncture was also performed the same day. The patient was transferred to the ICU due to concerns over airway protection due to rapidly worsening mental status. At this time a nine-panel drug screen was ordered which was negative. Lactic acid, procalcitonin, and blood cultures were all taken and were within normal limits. Nephrology was consulted for the hyponatremia, thought to be secondary to beer potomania and possible SIADH. In the ICU, the patient had a central line placed and he was started on 3% normal saline. Measurement of urine sodium was 191 mmol/L and urine osmolality was 626 mOsm/kg, although these labs were likely drawn after the administration of the hypertonic saline.

Initial results of the spinal tap showed a traumatic tap with 49 red blood cells/mm3, 8 white blood cells/mm3, glucose of 20 mg/dL, and protein of 188 mg/dL. Due to the low white blood cell count, ID felt meningitis was thought to be low on the differential. Acid fast culture was sent but would not return for several weeks from the health department. PCR for* Mycobacterium tuberculosis* was not available. A teleneurology consult was obtained due to the lack of an in-hospital neurology service, and they recommended treating the hyponatremia as a cause of the patient's encephalopathy. Due to the low white blood cell count in the cerebrospinal fluid, sterile blood cultures, and explanation of hyponatremia for the patient's altered mental status, antibiotics were discontinued on day #3 but eventually restarted as the patient's condition declined. He had a total of two weeks' course of antibiotics.

**Figure 1:**
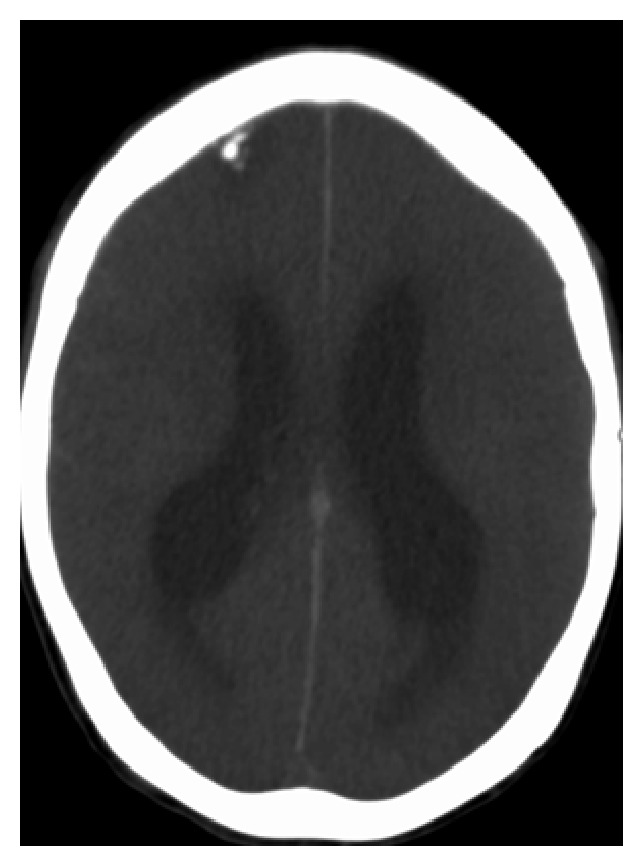
Head CT showing diffuse cerebral edema with ventriculomegaly.

**Figure 2:**
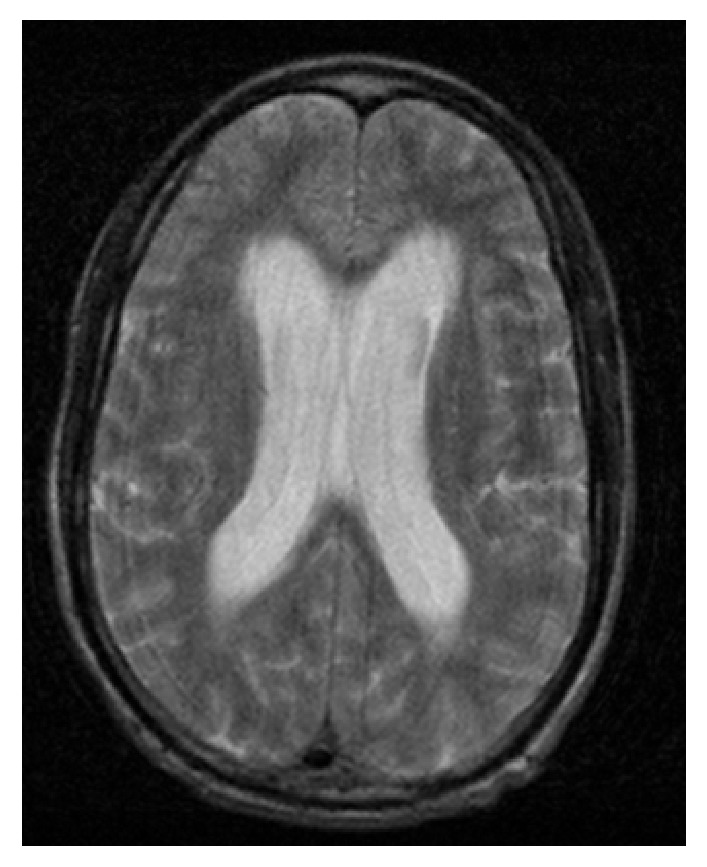
Head MRI showing moderate hydrocephalus and increased T2 flair between the gyri, concerning for meningitis.

Over the next two days the patient's mental status continued to wax and wane, as did his low grade fevers. Additional infection workup was ordered, including RPR, GC/chlamydia, hepatitis, and HIV, all of which were negative. Repeat chest X-ray showed worsening of the reticulonodular opacities previously visualized on the chest X-ray performed in the ED. A cardiac ECHO was ordered which was limited due to the patient developing tachycardia but showed normal EF and no signs of vegetations or valvular dysfunction. A repeat CT of the head was ordered as the patient's mental status continued to decline (Figure 1). CT of the head showed new onset hydrocephalus, leading to a consult being placed with neurosurgery. Due to the new findings on CT scan an MRI was ordered, which showed subtle increased T2/flair signal interdigitating between the gyri, concerning for leptomeningitis (Figure 2). Neurosurgery placed an external ventricular drain catheter to drain the cerebrospinal fluid and relieve the hydrocephalus. There was no elevated intracranial pressure. The neurosurgeon did raise concern for meningitis based on the abnormal results of the original cerebrospinal fluid studies and the MRI; however treatment for TB was not started due to the negative cerebrospinal fluid samples.

Daily cerebrospinal fluid counts were sent to the lab from the ventriculostomy fluid collected. These repeat cerebrospinal fluid samples showed elevated white blood cell count, elevated red blood cell count, and low glucose with elevated protein. In total, three acid fast cultures were sent to the lab for analysis but their results would not come out for some time from the Health Dept. Patient was continued on Vancomycin, Acyclovir, and Cefepime. Culture results did not return until almost one month later.

**Figure 3:**
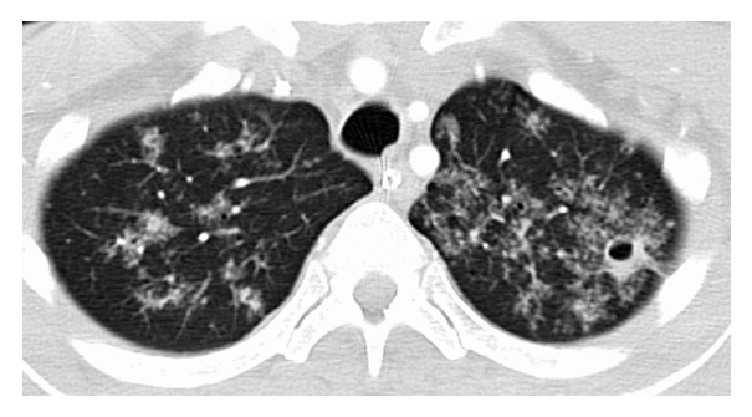
Chest CT scan showing diffuse airspace disease with associated reticular nodular opacities and apparent tree in bud configuration with small cavitary pulmonary nodule noted at the superolateral left lung apex.

Eight days into the patient's hospital stay and seven days into his ICU stay the patient experienced a cardiac arrest, likely due to increased pulmonary secretions and respiratory failure. He received ACLS with several rounds of epinephrine and suctioning and he eventually achieved return of spontaneous circulation. He was intubated for airway protection. A CT scan of the chest was ordered due to the persistently abnormal chest X-rays. CT of the chest showed diffuse airspace disease with associated reticular nodular opacities and apparent tree in bud configuration with small cavitary pulmonary nodule noted at the superolateral left lung apex (Figure 3). Bronchoalveolar lavage was performed by pulmonology to rule out infectious causes. Cultures were negative for fungus and grew out only normal respiratory flora. Either an acid fast culture was not sent from the bronchoalveolar washings or its results never came out at the Health Dept. Decision was made to transfer the patient to a local tertiary care hospital and the patient was accepted. However, no beds were available and the patient remained in critical condition in the ICU. A repeat MRI was performed which showed infarct of the left internal capsule and left putamen, as well as an acute small infarct in the right side of the pons. Neurosurgery continued to be concerned with infection including the possibility of TB. The ventricular drain shunt was discontinued as the intracranial pressure remained normal and the daily cerebrospinal fluid collection was stopped.

The patient began to experience worsening brain stem function during daily physical exams. QuantiFERON test was ordered and came back positive. This test was ordered due to the concern for extrapulmonary or meningeal TB from the prior chest CT which had showed a cavitary lesion. The patient was briefly started on four-drug RIPE therapy for only one day; however this was stopped as none of the acid fast cultures had yet returned positive. A cerebral perfusion test was ordered and was consistent with brain death. Family was contacted and present when the patient was extubated. He expired shortly after extubation.

## 3. Discussion/Literature Review

Our patient showed many of the classic signs and symptoms of meningeal TB, including a history of headache, worsening altered mental status, and abnormal cerebrospinal fluid protein and glucose values. In addition, MRI imaging of the brain and CT scan of the chest gave clues as to this patient's possible source of infection. Unfortunately, it would take several weeks for the results of the acid fast cerebrospinal fluid cultures to come out.

Review of the literature on MTB infections shows the onset of several stages of meningeal TB. Patients initially develop malaise and headache in the prodromal phase, which marks the entry of the bacteria into the cerebrospinal fluid. This stage is then followed by the meningitic and later the coma stages, all of which our patient progressed through [6]. Symptoms of the meningitic phase include altered mental status and fevers. Cerebrospinal fluid glucose levels are typically low (<40 mg/dL) while cerebrospinal fluid protein remains elevated (>100 mg/dL). A lymphocytic pleocytosis is also usually seen, with an elevated cerebrospinal fluid white blood cell count > 100/mm3.

Diagnosis is not required to begin treatment when clinical suspicion is high for TB as cultures can take several weeks to return. Newer cerebrospinal fluid PCR tests are available, with sensitivity and specificity approaching 90% [7]. Treatment consists of the standard RIPE regimen, including isoniazid, rifampin, pyrazinamide, and streptomycin or ethambutol [8].

## 4. Conclusion

It is critical to recognize the signs and symptoms of meningeal TB before the patient's condition worsens. Even general symptoms of headache with altered mental status should raise concerns for infectious causes. In our patient's case, the first lumbar puncture was performed 24 hours into the admission. The results were viewed differently by the various physicians taking care of the patient, and treatment for tuberculosis was started but never continued. The low white blood cell count in the cerebrospinal fluid was concluded to indicate a noninfectious etiology of the patient's altered mental status, even though the patient ended up growing MTB weeks later. Other prognostic indicators, including the positive QuantiFERON test and the abnormal CT scan, should have been considered. Skin PPD was never placed even though the CT scan showed a clearly well-defined cavitary lesion.

Other factors which contributed to the patient's death included an infarct which likely occurred from the tuberculous infection, poor history on presentation, and severe hyponatremia, which was never fully corrected back to normal sodium levels during the course of hospitalization.
